# UCP2-induced fatty acid synthase promotes NLRP3 inflammasome activation during sepsis

**DOI:** 10.1172/JCI169986

**Published:** 2023-03-15

**Authors:** Jong-Seok Moon, Seonmin Lee, Mi-Ae Park, Ilias I. Siempos, Maria Haslip, Patty J. Lee, Mijin Yun, Chun K. Kim, Judie Howrylak, Stefan W. Ryter, Kiichi Nakahira, Augustine M.K. Choi

Original citation: *J Clin Invest*. 2015;125(2):665–680. https://doi.org/10.1172/JCI78253

Citation for this retraction: *J Clin Invest*. 2023;133(6):e169986. https://doi.org/10.1172/JCI169986

Cornell University, Harvard Medical School, and Brigham and Women’s Hospital jointly notified the *JCI* that Figures 3A, 4D, 7B, and 8B and Supplemental Figures 4 and 7A are not reliable. In accordance with the institutional recommendations, the *JCI* is retracting this article.

## Version 1 03/15/2023

Electronic publication

